# Supplementary material for: A small signaling domain controls PPIP5K phosphatase activity in phosphate homeostasis
Source: Nat Commun. 2025 Feb 19;16:1753. doi: 10.1038/s41467-025-56937-0 (PMC11836120; doi:10.1038/s41467-025-56937-0)
Supplement: Supplementary file 1 — Supplementary Information [file 41467_2025_56937_MOESM1_ESM.pdf]

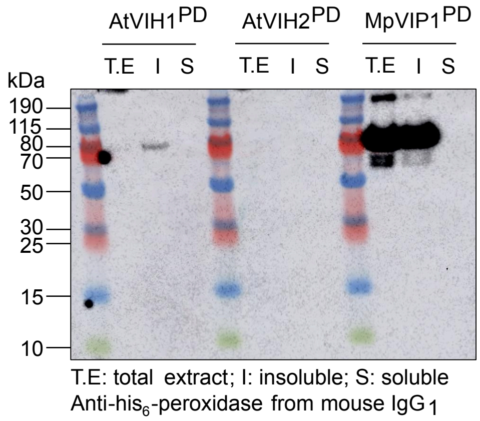

Fig. S1: Expression of AtVIH1^{PD}, AtVIH2^{PD} and MpVIP1^{PD} phosphatase domains in baculovirus-infected BTI-Tnao38 insect cells.
Western blot analysis showing the expression level and solubility of different PPIP5K phosphatase domain (PD) constructs from different plant species. The constructs analyzed are *Arabidopsis thaliana* (At) VIH1^{PD} (residues 351-1010, Uniprot ID Q84WW3), VIH2^{PD} (residues 351-1046, Uniprot ID F4J8C6), and *Marchantia polymorpha* (Mp) VIP1^{PD} (residues 382-1041 Uniprot ID Mp8g06840).

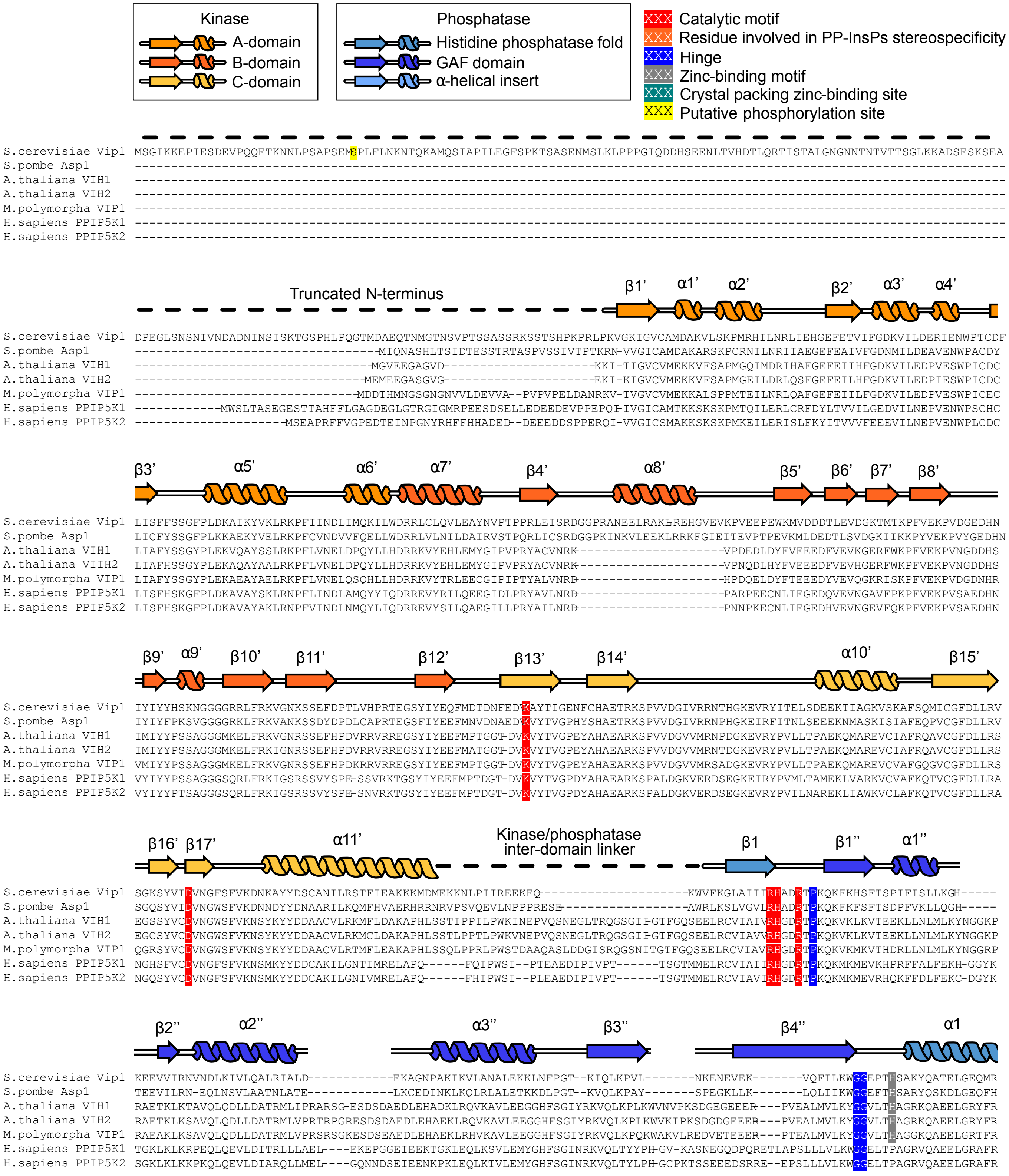

Kinase
A-domain
B-domain
C-domain
Phosphatase
Histidine phosphatase fold
GAF domain
α-helical insert
Catalytic motif
Residue involved in PP-InsPs stereospecificity
Hinge
Zinc-binding motif
Crystal packing zinc-binding site
Putative phosphorylation site
Truncated N-terminus
Kinase/phosphatase
inter-domain linker
S.cerevisiae Vip1
S.pombe Asp1
A.thaliana VIH1
A.thaliana VIH2
M.polymorpha VIP1
H.sapiens PPIP5K1
H.sapiens PPIP5K2

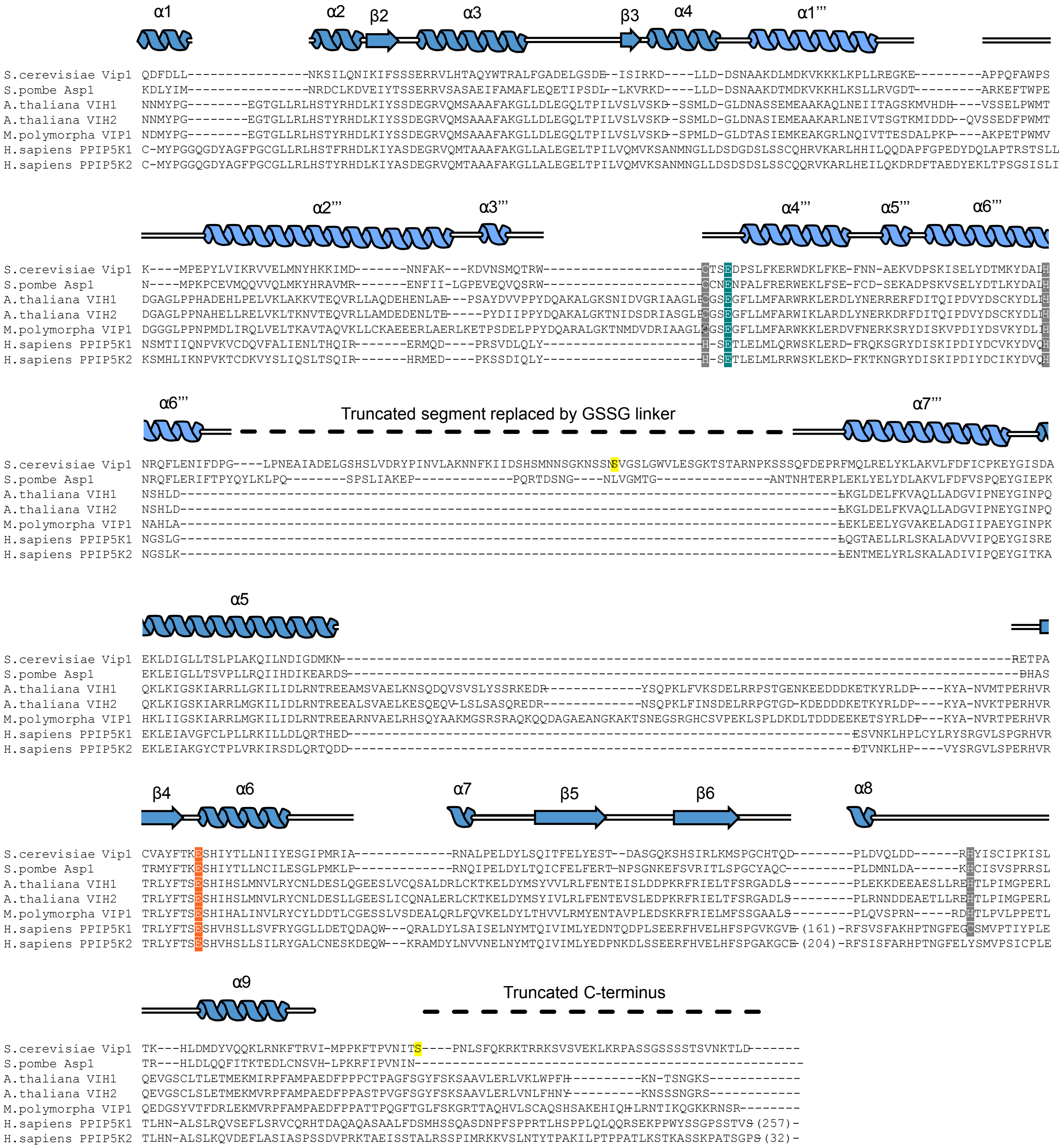

Fig. S2: Multiple sequence alignment of PPIP5K phosphatase domains.
Multiple-sequence alignment of *S. cerevisiae* Vip1 (Uniprot ID Q06685) with *Schizosaccharomyces pombe* Asp1 (Uniprot ID O74429), *Arabidopsis thaliana* VIH1 and VIH2 (Uniprot ID Q84WW3 and F4J8C6, respectively), *Marchantia polyorpha* VIP1 (Uniprot ID Mp8g06840), and *Homo sapiens* PPIP5K1 and PPIP5K2 (Q6PFW1 and O43314, respectively). Secondary structure elements are colored according to different domains as indicated on top. Functionally important conserved residues are highlighted. Dashed lines indicate regions of the protein truncated in the engineered constructs of ScVip1 kinase domain (residues 186-522) and ScVip1 phosphatase domain (residues 536-1107, Δ848-918) used for crystallization

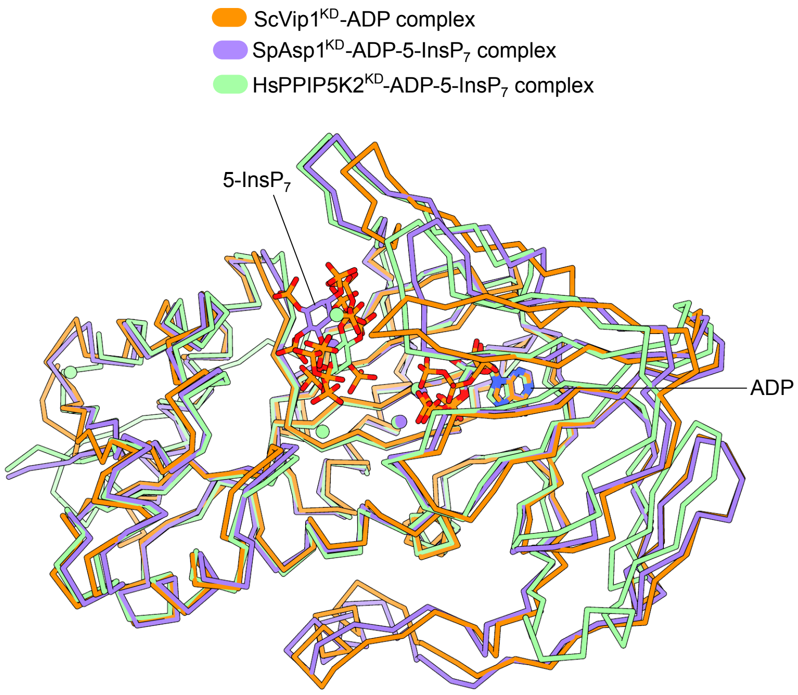

Fig. S3: ScVip1 harbors a structurally conserved PPIP5K kinase domain.
Structural comparison of different PPIP5K kinase domain structures. Shown are Cα traces of the *Saccharomyces cerevisiae* Vip1 kinase domain (in orange, bound to ADP in bonds representation), *Homo sapiens* PPIP5K2 (PDB id: 3T9E, in green and bound to ADP and 5-InsP$_7$) (Wang et al, 2012) and *Schizosaccharomyces pombe* Asp1 (PDB id: 8E1H, in purple) (Benjamin et al, 2022b). The kinase structures align with a r.m.s.d of ~1.5 Å comparing 310 Cα atoms.

a

PDB id	Z	Description
1ND6	21.5	Prostatic acid phosphatase (*H. sa*)
3IT2	21.4	Histidine acid phosphatase (*F. tu*)
…	…	…
1NT4	20.2	Periplasmic glucose-1 phosphatase (*E. co*)
1DKL	20.0	Phytase (*E. co*)
…	…	…
4JOC	18.8	Lysophosphatidic acid phosphatase type 6 (*H. sa*)
5CX4	18.8	Inositol polyphosphate phosphatase (*B. th*)
…	…	…
3EOZ	12.8	Phosphoglycerate mutase (*P. fa*)
3DCY	12.5	TIGAR (TP53 induced glycolysis and apoptosis regulator) (*H. sa*)
1UJC	12.4	SixA histidine phosphatase (*E. co*)
5XD2	12.3	Nudix MutT1 (*M. sm*)
3E9E	12.3	TIGAR fructose-2,6- and fructose-1,6-bisphosphatase (*D. re*)
3O0T	12.1	Ser/Thr phosphatase PGAM5 (phosphoglycerate mutase 5) (*H. sa*)
5UM0	11.9	2,3-bisphosphoglycerate-dependent phosphoglycerate mutase (*N. go*)
…	…	…
5ZR2	11.4	Phosphoserine phosphatase (*E. hi*)
1EBB	11.3	Cofactor-dependent phosphoglycerate mutase homolog PhoE (or YhfR) (*B. st*)
…	…	…
6NRU	10.9	Alpha-ribazole phosphatase (*S. fl*)
…	…	…
3D6A	9.9	2H-phosphatase domain of Sts-2 (*M. mu*)
…	…	…
1K6M	8.2	Liver PFKFB (6-phosphofructo-2-kinase/Fructose-2,6-bisphosphatase) (*H. sa*)
…	…	…
3PGM	7.6	Phosphoglycerate mutase (*S.ce*)
…	…	…

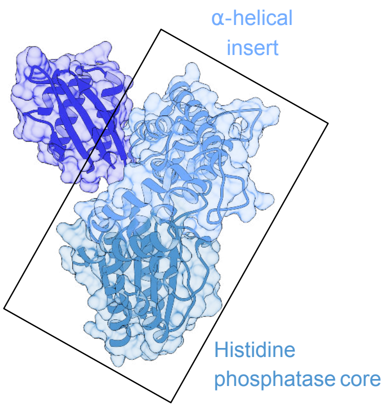

b

PDB id	Z	Description
6FWS	5.9	Arch domain of the ATP-dependent DNA helicase DinG (*E. co*)
7zsa	5.8	RNA polymerase II subunit RPB1 (*S. ce*)
7O4K	5.5	Arch domain of the transcription factor IIH helicase subunit (*S. ce*)
…	…	…
3R5A	4.6	Tetrahydrodipicolinate N-succinyltransferase (*P. ae*)
…	…	…
5N0L	4.3	GAF domain from the nutrient sensor CodY (*C. di*)
3IBJ	4.3	GAF domain from the phosphodiesterase 2A (*H. sa*)
4JGO	4.2	PAS-like domain from the sporulation kinase D (*B. su*)
3A1J	4.2	Rad9 subunit from the Rad9-Hus1-Rad1 DNA sliding clamp (*H. sa*)
…	…	…
3EEH	4.0	Putative light and redox sensing histidine kinase (*H. ma*)
7JTK	4.0	GAF domain from RSP2 subunit of the flagellar radial spoke 1 (*C. re*)
…	…	…
8W7P	3.9	PAS domain from a sensor histidine kinase (*P. sp. FPU-7*)
…	…	…
5IJJ	3.8	SPX domain from a glycerophosphodiester phosphodiesterase 1 (*T. th*)
…	…	…
4MCW	3.8	GAF domain from a cyclic-di-GMP phosphodiesterase (*P. ma. EX-H1*)
…	…	…
5W10	3.8	GAF domain from a cAMP-dependent diguanylate cyclase (*L. in*)
…	…	…
6HMJ	3.6	PAS domain from a photoreceptor PAL (*N. mu. DSM 44233*)
…	…	…
8UFI	3.6	GAF domain from a phosphodiesterase 6 (*B. ta*)
8HXX	3.6	Transcriptional regulatory protein SIN3 (*S. ce*)
…	…	…

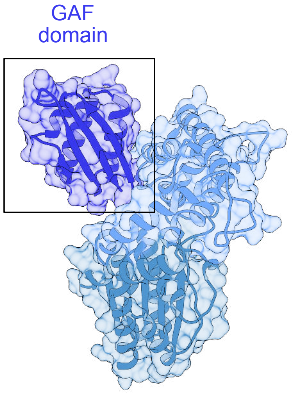

Fig. S4: ScVip1[PD] is structurally related to histidine acid phosphatases and contains a GAF domain.
Top twenty hits from the DALI web server (http://ekhidna2.biocenter.helsinki.fi/dali/) (Holm & Rosenström, 2010) using the histidine acid phosphatase core containing the α-helical insertion domain (residues 536-553; 647-1094) (**a**), or the isolated GAF domain in ScVip1[PD] (residues 554-646) (**b**) as search model against the Protein Data Bank.

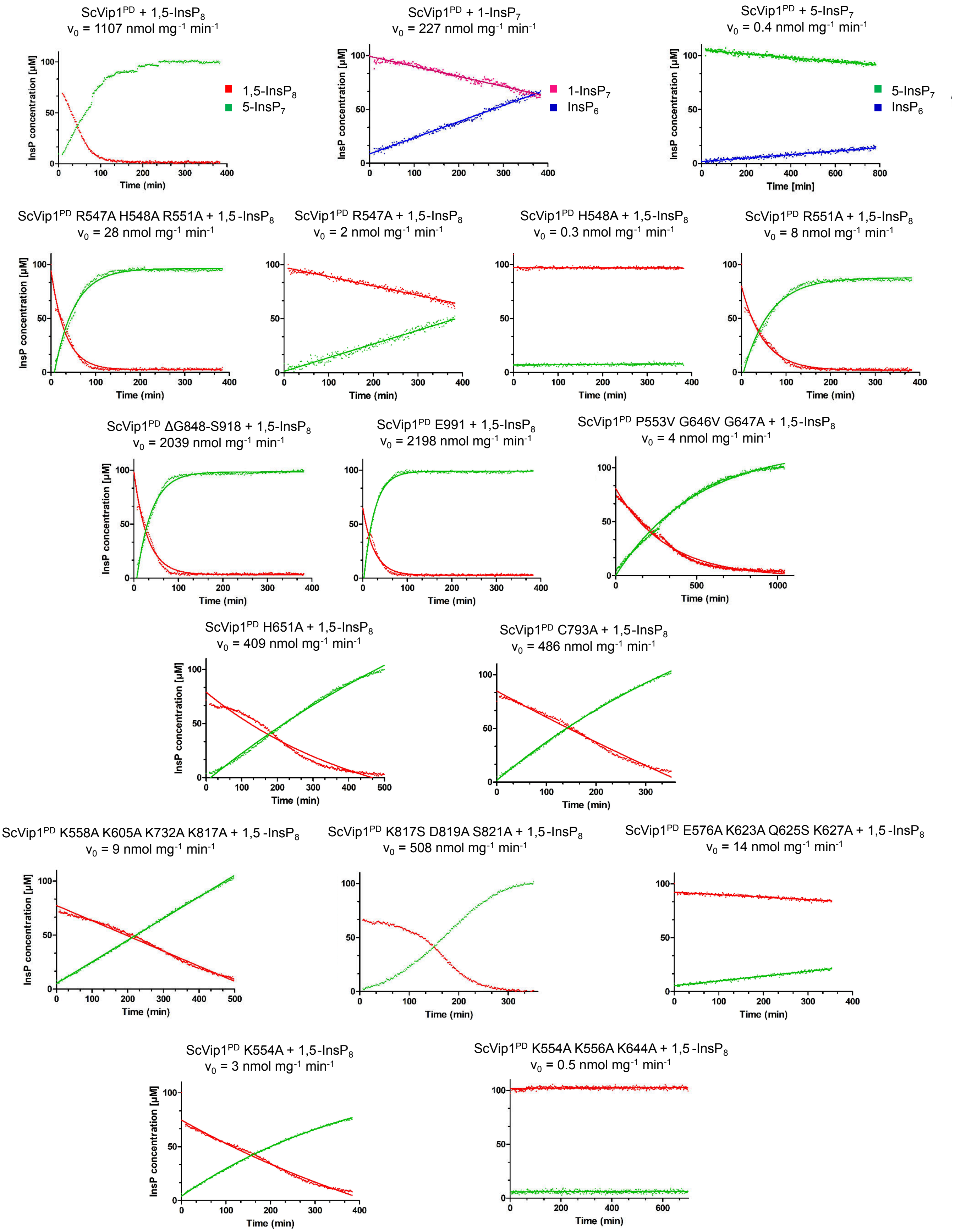

Fig. S5: NMR-based enzyme assays – raw data.
NMR time course experiments of PP-InsP hydrolysis catalyzed by ScVip1^{PD} and respective mutant constructs using 100µM of [13C6]-labeled PP-InsP substrates. Concentrations of enzyme were varied based on the respective construct and substrate. PP-InsP turnover was measured using a pseudo-2D spin-echo difference nuclear magnetic resonance spectroscopy experiment. Quantification was based on the relative intensities of the C2 signals of the respective (PP-)InsP species.

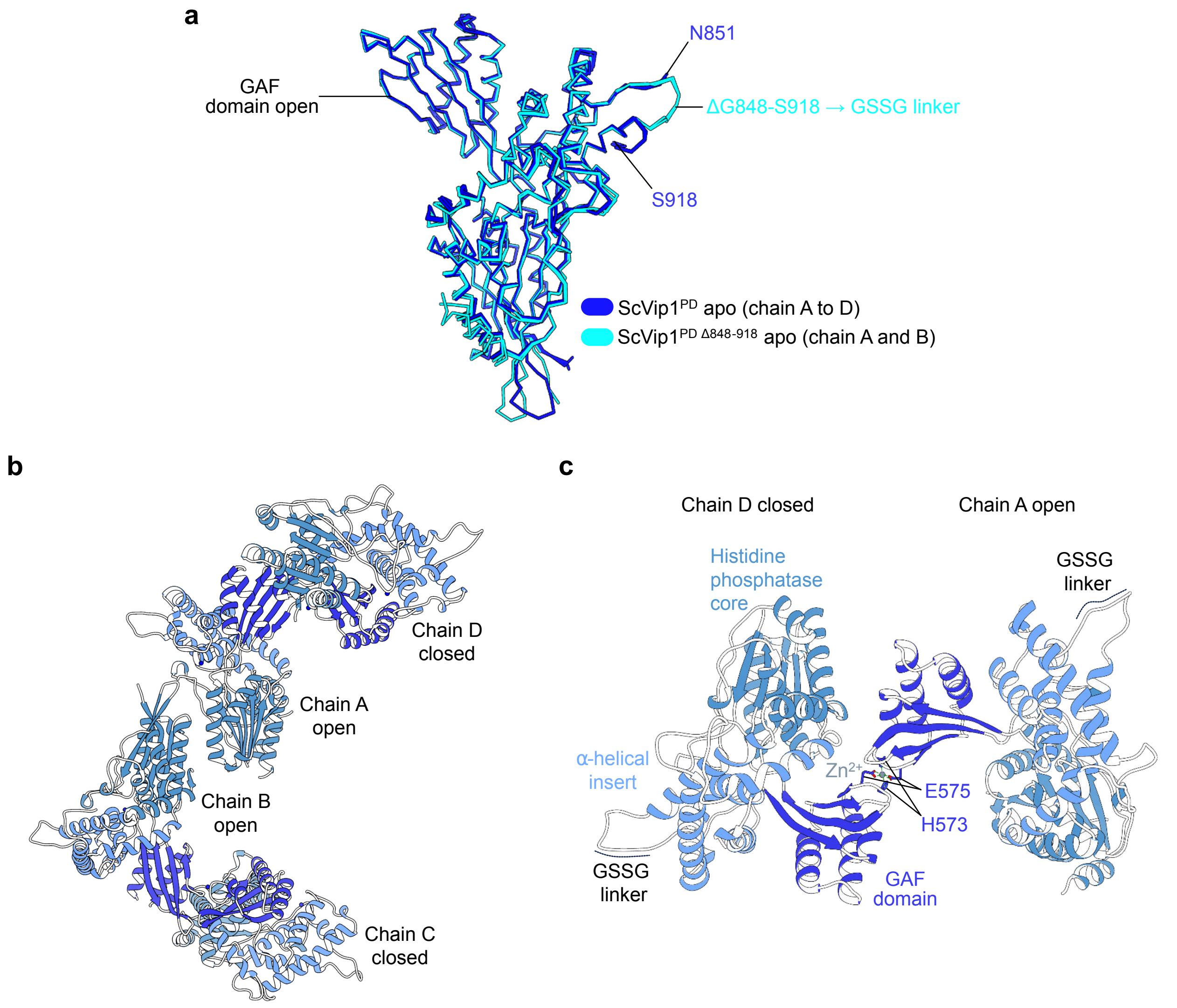

Fig. S6: Arrangement of GAF domains in the ScVip1^{PD} and ScVip1$^{PD\ \Delta848\text{-}918}$ apo structures.

a Structural superposition of chains A (shown as Cα traces) from the ScVip1^{PD} (in blue) and ScVip1$^{PD\ \Delta848\text{-}918}$ (in cyan) apo crystal forms reveal the GAF domain in an open conformation (r.m.s.d is ~0.5 Å comparing 457 corresponding Cα atoms). The asymmetric unit of ScVip1^{PD} apo contains four molecules, all of which present the GAF domain in the open conformation. **b** ScVip1$^{PD\ \Delta848\text{-}918}$ crystals also contain four molecules in the asymmetric unit with chains A and B in the open and chains C and D in the closed conformation, respectively. Shown are ribbon diagrams, colors as in Fig. 1b. **c** A second Zn^{2+} binding site in ScVip1$^{PD\ \Delta848\text{-}918}$ is formed along a pseudo 2-fold axis involved two neighboring GAF domains. This second binding site likely represent a crystallization artifact. The crystallization condition contained an excess of $(CH_3CO_2)_2Zn$ and the residues involved in Zn^{2+} binding are not conserved among other PPIP5Ks (His573, Glu575, compare Fig. S2).

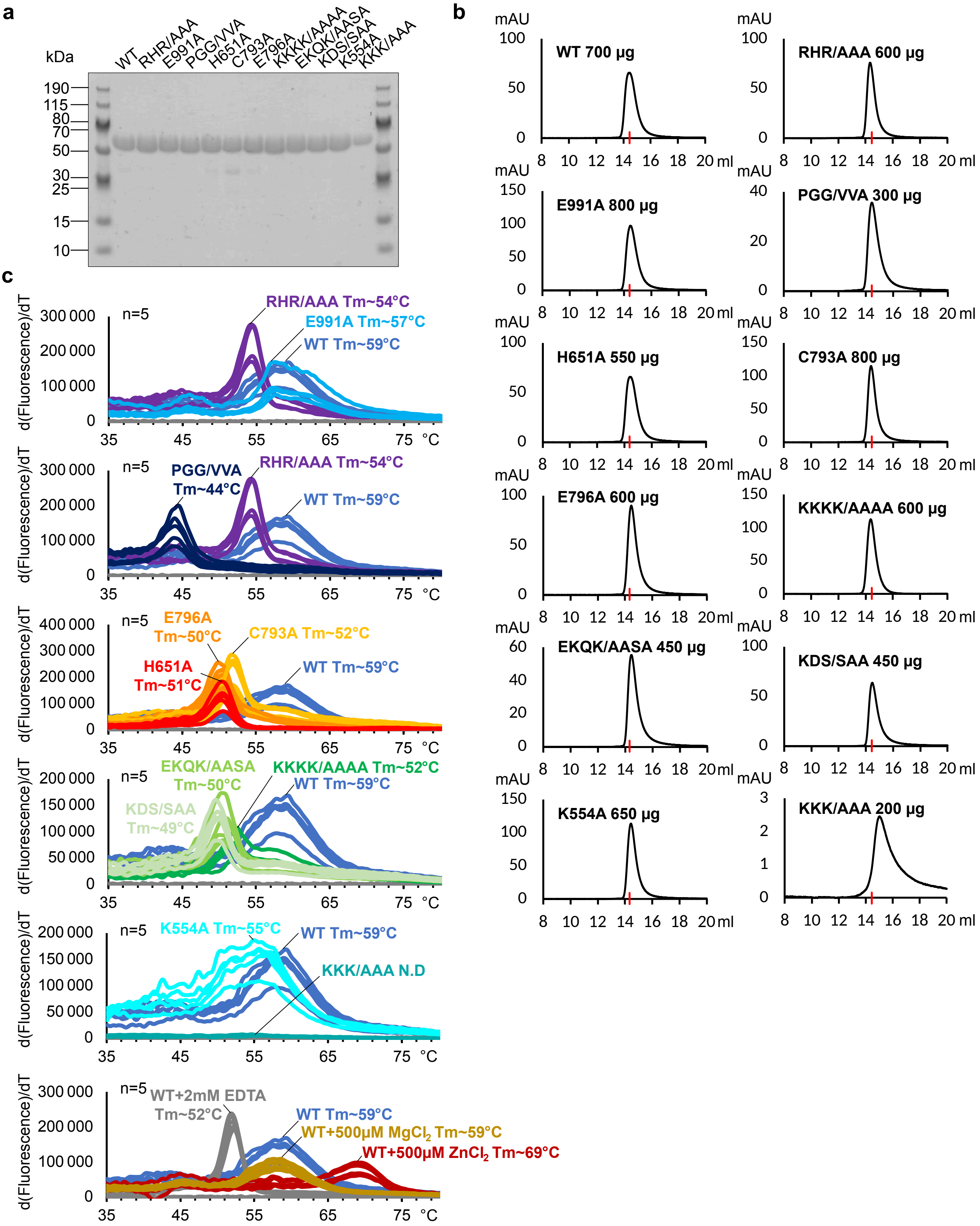

Fig. S7: Structural integrity profiling of ScVip1^{PD} mutant proteins.
a Coomassie blue-stained SDS-PAGE analysis of wild-type and mutant versions of ScVip1^{PD} (theoretical molecular weight is ~65.5 kDa). WT: ScVip1^{PD}, RHR/AAA: ScVip1$^{PD\ R547A\text{-}H548A\text{-}R551A}$, PGG/VVA: ScVip1$^{PD\ P553V\text{-}G646V\text{-}G647A}$, KKKK/AAAA: ScVip1$^{PD\ K558A\text{-}K605A\text{-}K732A\text{-}K817A}$, EKQK/AASA: ScVip1$^{PD\ E576A\text{-}K623A\text{-}Q625S\text{-}K627A}$, KDS/SAA: ScVip1$^{PD\ K817S\text{-}D819A\text{-}S821A}$, KKK/AAA: ScVip1$^{PD\ K554A\text{-}K556A\text{-}K644A}$. **b** Analytical size-exclusion chromatography of wild-type and mutant versions of ScVip1^{PD}. Shown are A_{280nm} absorption traces as a function of column elution volume (in mL). The red arrow indicates the characteristic elution volume of ScVip1^{PD}, which behaves as a monomer in solution. **c** Melting temperatures of wild-type (blue traces, n=5) and mutant versions of ScVip1^{PD} derived from thermal shift assays (N.D. not determined). Note that addition of divalent metal chelators such as EDTA reduces the thermal stability of the enzyme. Addition of $ZnCl_2$ but not $MgCl_2$ increases ScVip1^{PD} stability, likely by targeting the structural Zn^{2+} binding site.

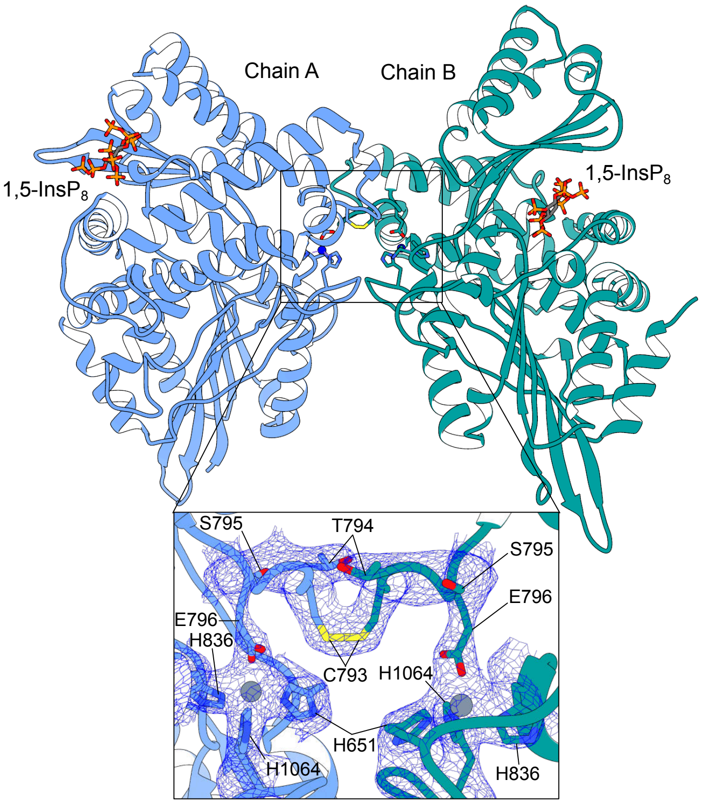

Fig. S8: An intermolecular disulfide bridge stabilizes the crystallographic dimer in the ScVip1$^{PD\ \Delta848\text{-}918\ RHR\text{-}AAA}$ – 1,5-InsP$_8$ complex structure.

Ribbon diagram of a crystallographic domain-swapped dimer in the asymmetric unit of our ScVip1$^{PD\ \Delta848\text{-}918\ RHR\text{-}AAA}$ crystals (chain A is shown in blue, chain B in green, 1,5-InsP$_8$ molecules are depicted in bonds representation). The inlet provides a detailed view of the Zn^{2+} binding site, with the Zn^{2+} ions shown as a sphere (in gray) and the coordinating residues depicted in bonds representation. The view includes a 2 (Fo – Fc) electron density map contoured at 1 σ (blue mesh). Cys793 is involved in an intermolecular disulfide bridge between ScVip1$^{PD\ \Delta848\text{-}918\ RHR\text{-}AAA}$ chain A (in blue) and chain B (in green). In the crystal structure of the apo ScVip1$^{PD\ \Delta848\text{-}918}$ (compare Fig1C), Cys793 replaces Glu796 and coordinates the Zn^{2+} ion.

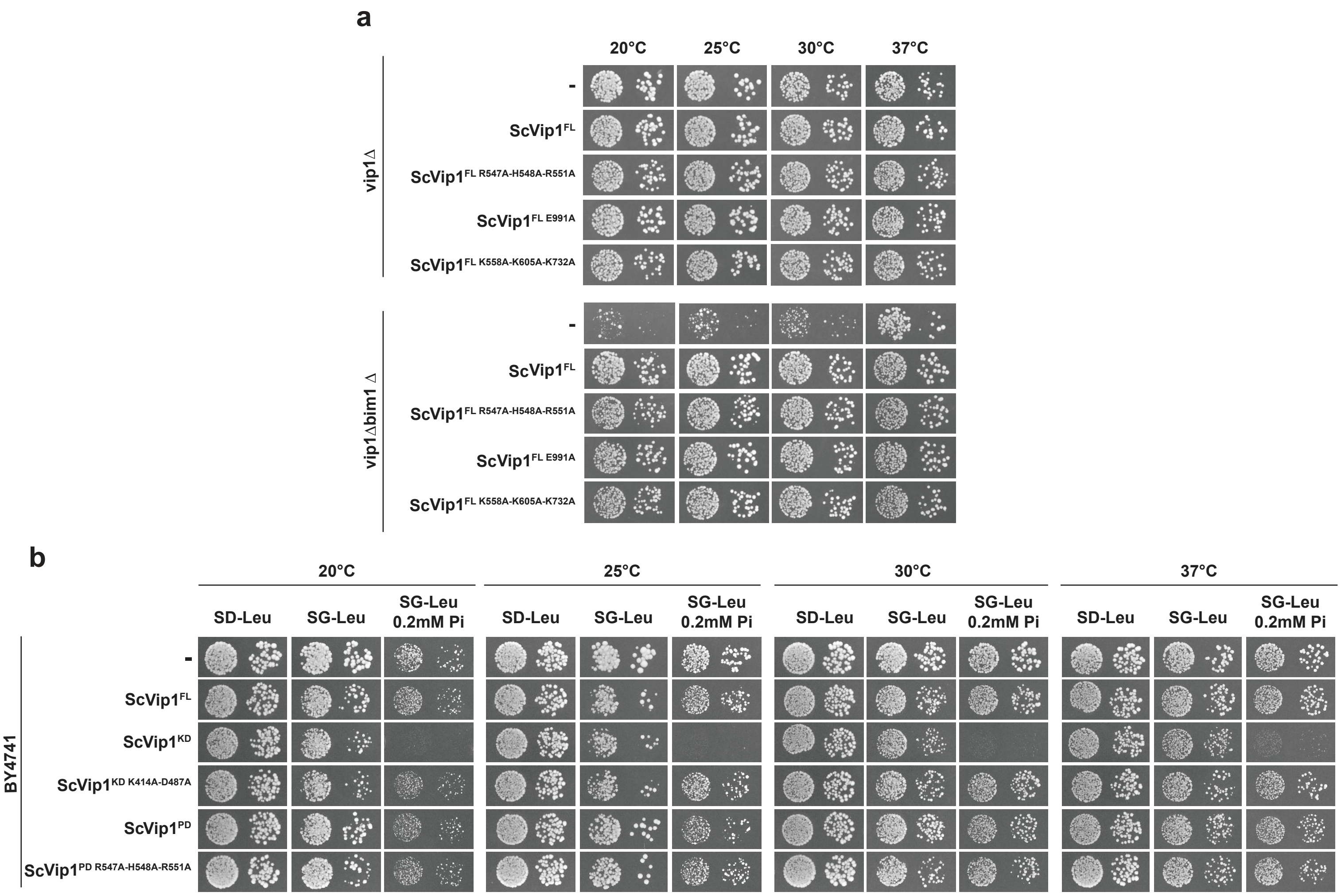

Fig. S9: Genetic studies of Vip1 kinase and phosphatase domains in budding yeast.
a The depicted yeast vip1Δ and vip1Δbim1Δ strains expressing empty vector (-), ScVip1^{FL} (full-length) or ScVip1^{FL} mutants were spotted in 10-fold serial dilutions on glucose- and galactose-containing medium at standard and low concentrations of potassium phosphate (0.2 mM Pi) and incubated at the indicated temperatures for 2–11 days. **b** BY4741 strain expressing empty vector (-), ScVip1^{FL}, ScVip1^{KD} (186-524), ScVip1^{PD} (536-1107) or mutants (as indicated) were spotted in 10-fold serial dilutions on glucose-, galactose-containing medium at standard and low concentrations of potassium phosphate (0.2 mM Pi) and incubated at the indicated temperatures for 2–11 days.

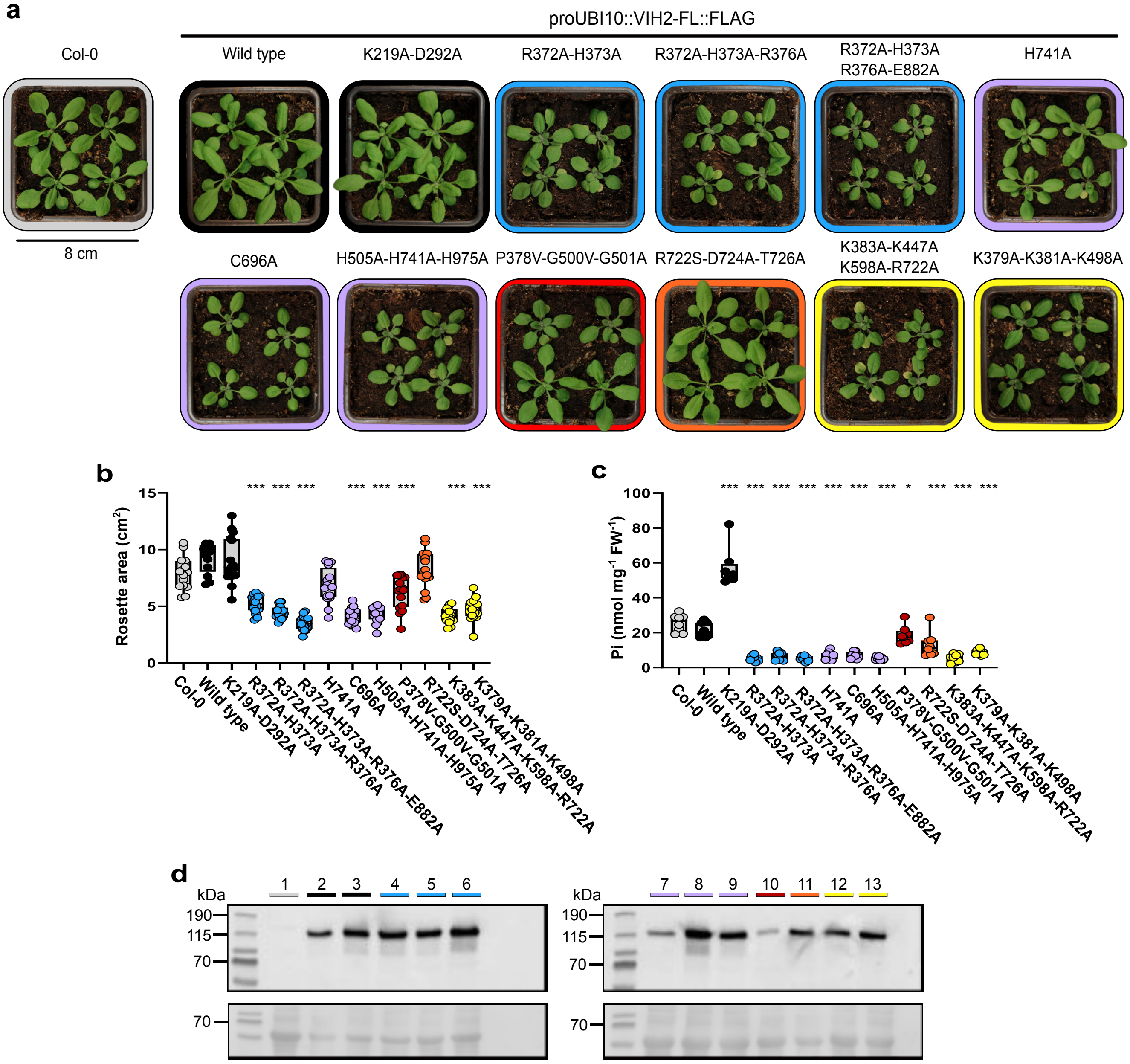

Fig. S10: Structure-function analysis of the AtVIH2 phosphatase domain – independent replication experiment.
a Rosette phenotype of three weeks old Col-0 wild-type plants and lines expressing full-length Flag-tagged versions of AtVIH2 under the control of the UBI10 promoter. **b** Quantification of the rosette area. Box plots show 16 plants per genotype (n=16). Multiple comparisons of the genotypes vs. Col-0 were performed according to Dunnett[102] as implemented in GraphPad prism v10.3.0 (*** $p < 0.001$, ** $p < 0.005$, * $p < 0.01$). **c** Quantification of cellular Pi levels. For each genotype, 8 individual plants were measured using 3 technical replicates (n=8). The estimated Pi concentration was normalized by fresh weight. **d** Western blot of Flag-tagged AtVIH2. The theoretical molecular mass of AtVIH2 is ~118 kDa (indicated by a black arrow; 1: Col-0, 2: AtVIH2^{WT}, 3: AtVIH2$^{K219A\text{-}D292A}$, 4: AtVIH2$^{R372A\text{-}H373A}$, 5: AtVIH2$^{R372A\text{-}H373A\text{-}R376A}$, 6: AtVIH2$^{R372A\text{-}H373A\text{-}R376A\text{-}E882A}$, 7: AtVIH2^{H741A}, 8: AtVIH2^{C696A}, 9: AtVIH2$^{H505A\text{-}H741A\text{-}H975A}$, 10: AtVIH2$^{P378V\text{-}G500V\text{-}G501A}$, 11: AtVIH2$^{R722S\text{-}D724A\text{-}T726A}$, 12: AtVIH2$^{K383A\text{-}K447A\text{-}K598A\text{-}R722A}$, 13: AtVIH2$^{K379A\text{-}K381A\text{-}K498A}$.

a

Description	ScVip1^{KD-PD}	ScVip1^{KD-PD} + 1,5-InsP$_8$	ScVip1^{KD-PD} + AMPPNP + 1,5-InsP$_8$
Reaction volume	50 µl	50 µl	50 µl
% D_2O in the reaction	77,0%	77,0%	77,0%
Temperature	22 °C	22 °C	22 °C
D_2O incubation times (sec)	30 ; 300	30 ; 300	30 ; 300
Control sample	Non-deuterated (ND) ScVip1		
Quench buffer (20 µl)	4M Gdn-HCl, 1M NaCl, 0.1M NaH_2PO_4 (pH 2.5), 1% Formic Acid (FA)		
Number of peptides anayzed	121	121	121
Sequence coverage	97,0%	97,0%	97,0%
Replicates	3 ; 3	3 ; 3	2 ; 2
Criteria for HDX rate difference	Difference of HDX level at a given timepoint is > 7 % and > 0.5 Da and p values of student t-test is < 0.02		
Protein amount (nmol)	0,107	0,107	0,107
Ligand amount (nmol)	-	10,71	10.71 + 10.71

b

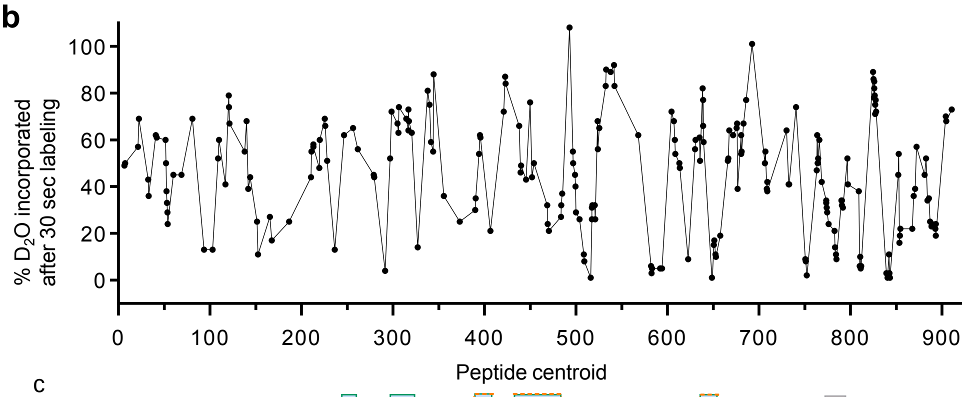

c

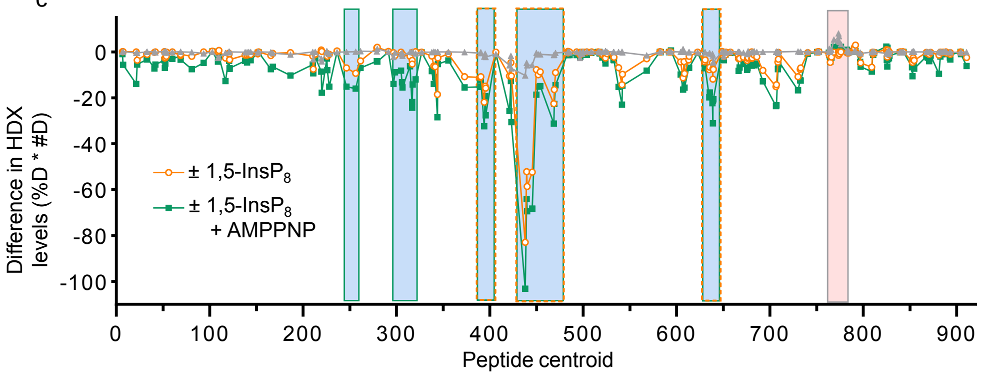

Fig. S11: Summary of the HDX-MS experiments

a Experimental details of the HDX analyses. **b** D2O incorporation into ScVip1^{KD-PD}. **c** HDX differences comparing apo ScVip1^{KD-PD}, ScVip1^{KD-PD} in complex with 1,5-InsP$_8$ and ScVip1^{KD-PD} in the presence of 1,5-InsP$_8$ and a non-hydrolyzable ATP analog (blue, protection; red, deprotection).

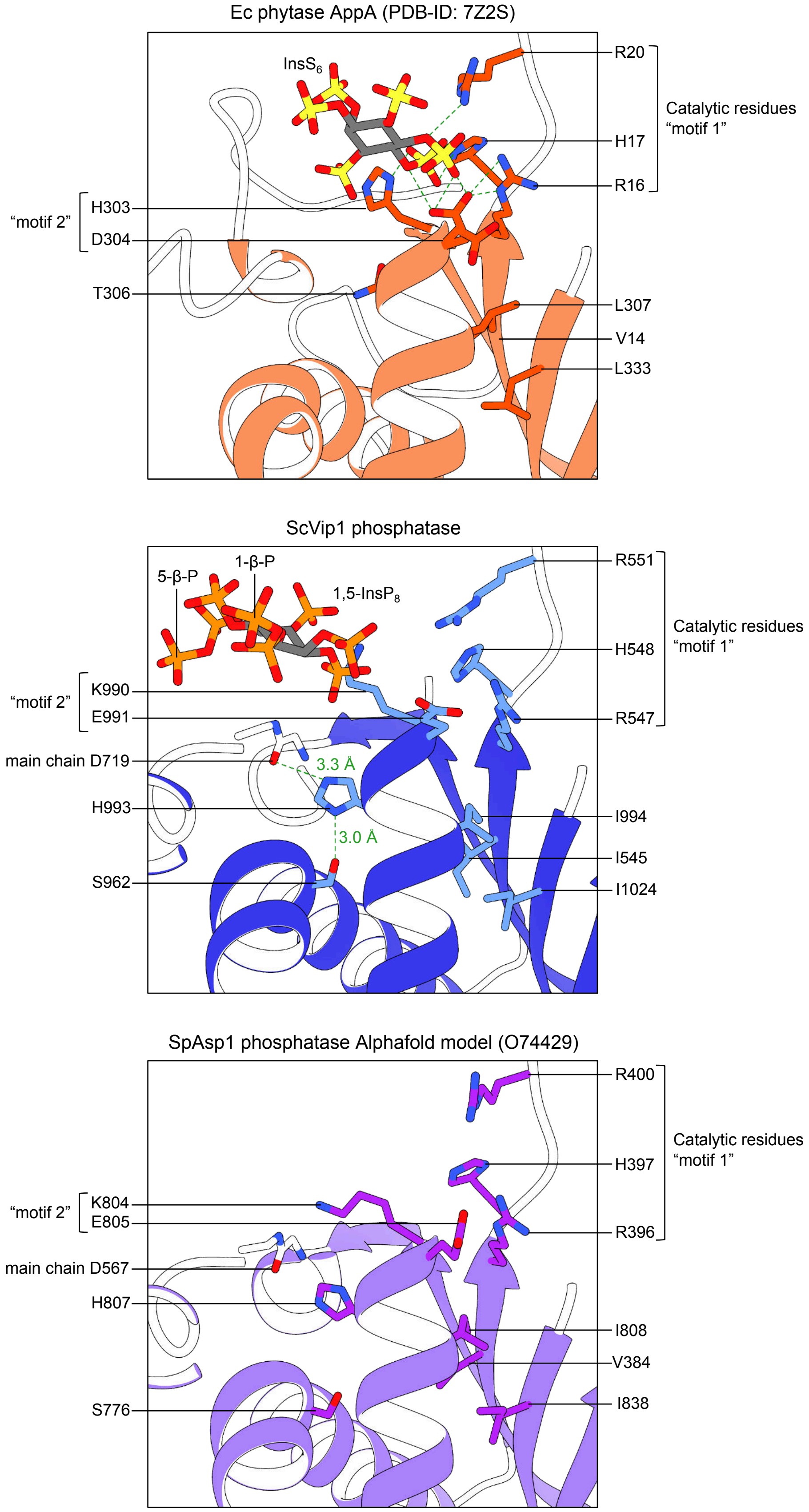

Fig. S12: The "HD" catalytic motif presents in phytases is not present in ScVip1^{PD}.
Detailed views of the active sites of *E. coli* phytase AppA (in orange, PDP-ID 7Z2S) (Acquistapace et al, 2022) bounds to the substrate analogue $InsS_6$, *S. cerevisiae* Vip1$^{PD\ \Delta848\text{-}918}$, and the Alphafold model of *S. pombe* Asp1^{PD} (in purple, https://alphafold.ebi.ac.uk/entry/O74429) (Varadi et al, 2024). The $InsP_6$ and 1,5-$InsP_8$ molecules are depicted in bonds representation, and the side chains of residues belonging to the catalytic motifs 1 and 2 are depicted bonds representation. Residues His303 and Asp304 forming the HD motif in AppA correspond to Lys990 and Glu991 in ScVip1^{PD} and not to His993 and Ile994, as previously thought (Fridy et al, 2007; Mulugu et al, 2007; Pöhlmann et al, 2014; Wang et al, 2015a; Pascual-Ortiz et al, 2018).

PDB-ID	**ScVip1^{KD} ADP** **9GR8**	**ScVip1^{PD} Pt MR-SAD**	**ScVip1^{PD} apo** **9GRH**	**ScVip1$^{PD\Delta 848\text{-}918}$ apo** **9GRN**	**ScVip1$^{PD\ \Delta 848\text{-}918}_{RHR\text{-}AAA}$ 1,5-InsP$_8$** **9GRO**
**Data collection**					
Wavelength	1.000036	1.071846	0.999993	1.000031	1.000000
Space group	$P\ 2_1$	P 1	$P\ 2_1$	C 2	$P\ 3_1\ 2\ 1$
Cell dimensions					
a, *b*, *c* (Å)	40.7, 83.2, 51.8	84.5, 84.7, 101.1	84.4, 194.9, 84.4	249.5, 90.5, 200.9	114.6, 114.6, 172.2
α, β, γ (°)	90, 94.32, 90	86.6, 74.1, 86.6	90, 112.2, 90	90, 126.91, 90	90, 90, 120
Resolution (Å)	43.86 – 1.18 (1.25 – 1.18)	48.55 – 3.50 (3.71 – 3.50)	48.7 – 3.05 (3.23 – 3.05)	45.26 – 3.40 (3.60 – 3.40)	49.68 – 2.36 (2.51 – 2.36)
R_{meas}#	0.104 (2.02)	0.106 (1.04)	0.158 (1.71)	0.46 (2.70)	0.110 (2.94)
CC(1/2)#	1.0 (0.55)	1.0 (0.40)	1.0 (0.48)	0.98 (0.30)	1.0 (0.52)
$I/\sigma I$#	13.3 (1.2)	7.2 (1.0)	10.1 (1.2)	5.2 (1.0)	19.6 (1.0)
Completeness (%)#	97.5 (93.4)	95.2 (95.3)	99.7 (99.8)	99.0 (98.5)	99.9 (99.6)
Redundancy#	6.7 (6.2)	1.8 (1.9)	7.0 (7.2)	6.9 (7.2)	20.4 (19.7)
Wilson B-factor#	18.3	111.3	83.8	72.2	73.2
**Phasing**					
No. sites (Pt/Zn)		4/4			
Figure of merit*		0.64			
**Refinement**					
Resolution (Å)	43.86 – 1.18		48.7 – 3.05	45.26 – 3.50	49.68 – 2.36
No. reflections	215,320		48,029	49,444	54,125
Twin operator / fraction+			l, -k, h / 0.5		
R_{work} / R_{free}+	0.15 / 0.18		0.267 / 0.31	0.24 / 0.29	0.22 / 0.26
No. atoms					
protein	2,845		15,722	15,461	7,819
ligand	47		4	255	241
solvent	397			46	51
Res. B-factors+					
protein	19.8		120.1	101.1	91.1
ligand	28.3		113.1	113.9	110.3
solvent	34.7			57.3	74.3
R.m.s deviations$					
bond lengths (Å)	0.021		0.002	0.006	0.003
bond angles (°)	1.81		0.52	1.0	0.53
Ramachandran plot$:					
most favored regions (%)	98.2		95.8	96.7	96.7
outliers (%)	0		0.5	0.1	0.0
MolProbity score$	1.21		1.84	2.18	1.30

#as defined in XDS[77]

*as defined in PHASER-EP[81]

+as defined in phenix.refine[80]

$as defined in Molprobity[86]

Table S1: Crystallographic data collection and refinement statistics.

Uncropped gel, shown in Supplementary Fig. 10

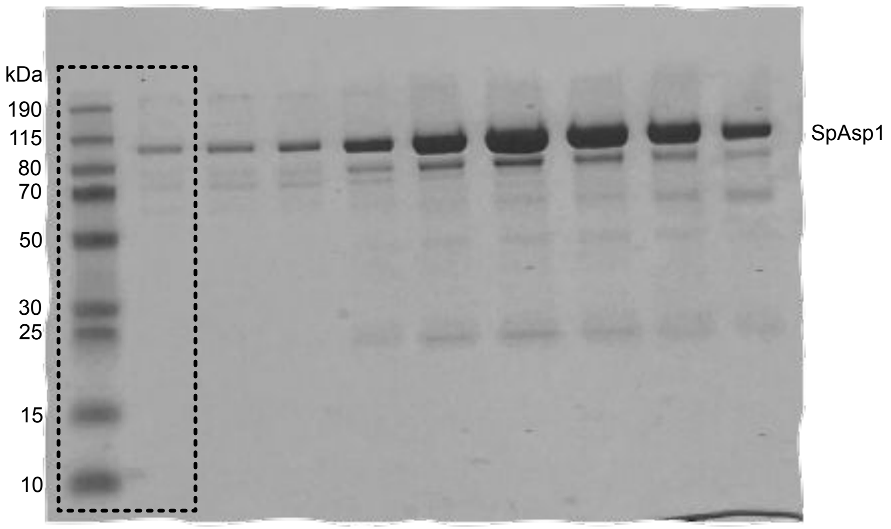

Uncropped membrane, shown in Supplementary Fig. S1

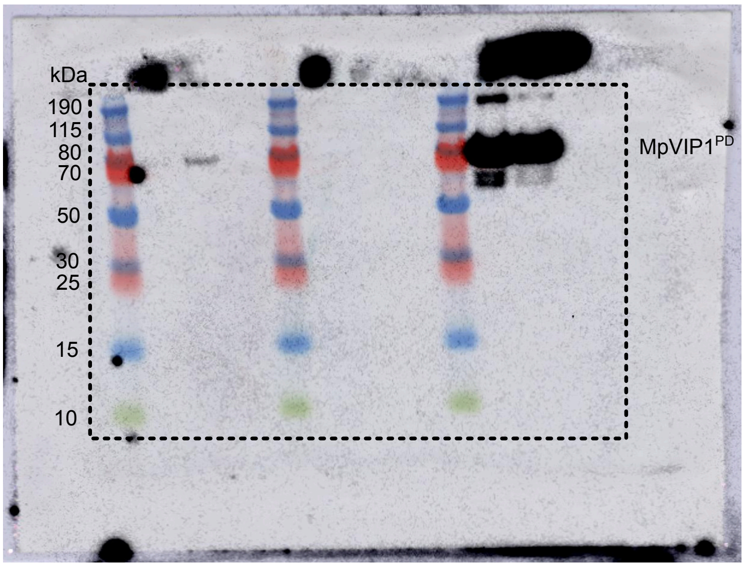

Anti-his6-peroxidase from mouse IgG 1 (dilution 1:3000)

Uncropped membrane in Fig. 9e

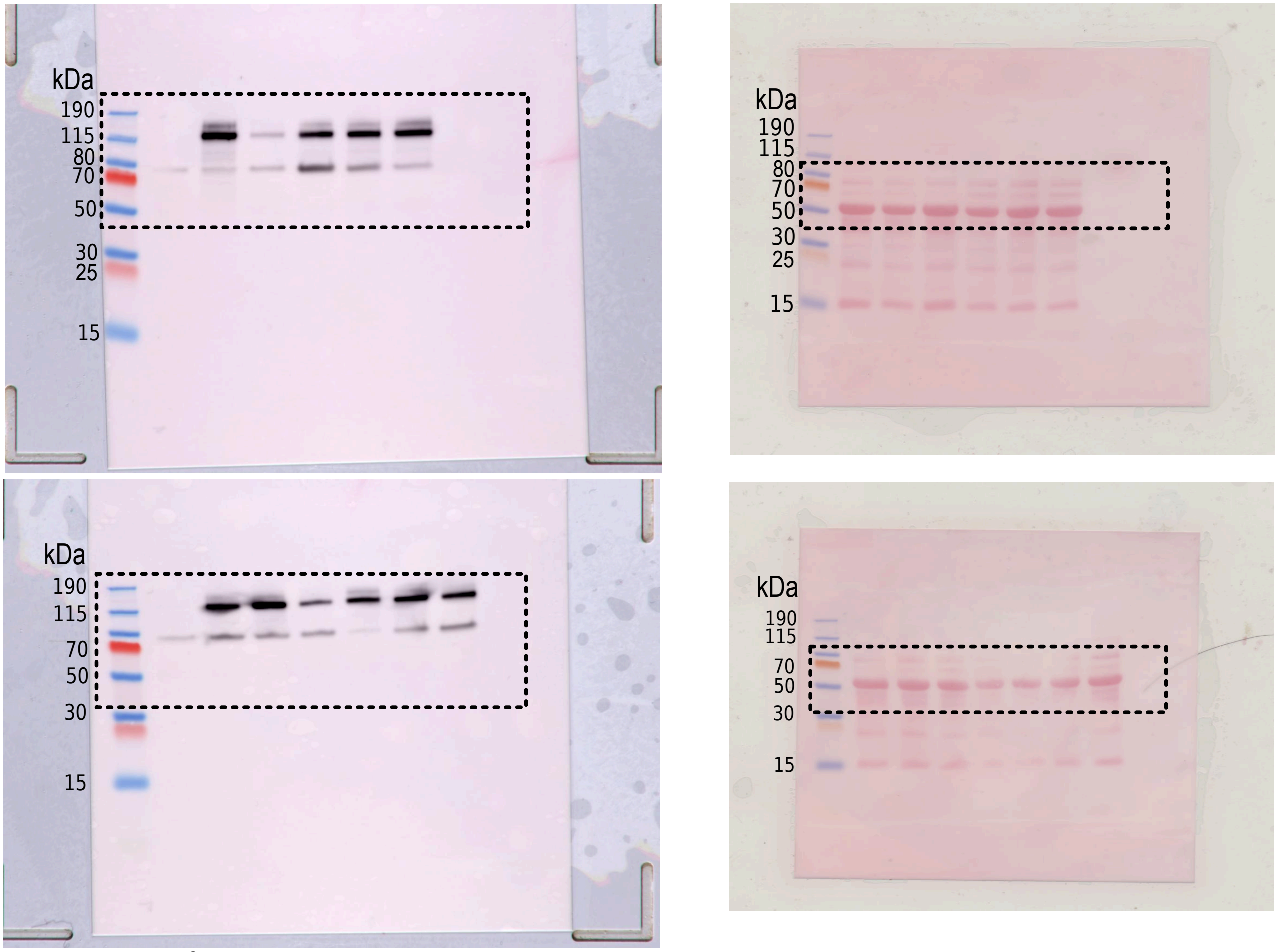

Monoclonal Anti-FLAG-M2-Peroxidase (HRP) antibody (A8592, Merck) (1:5000)

Uncropped membrane in Supplementary Fig. S10d

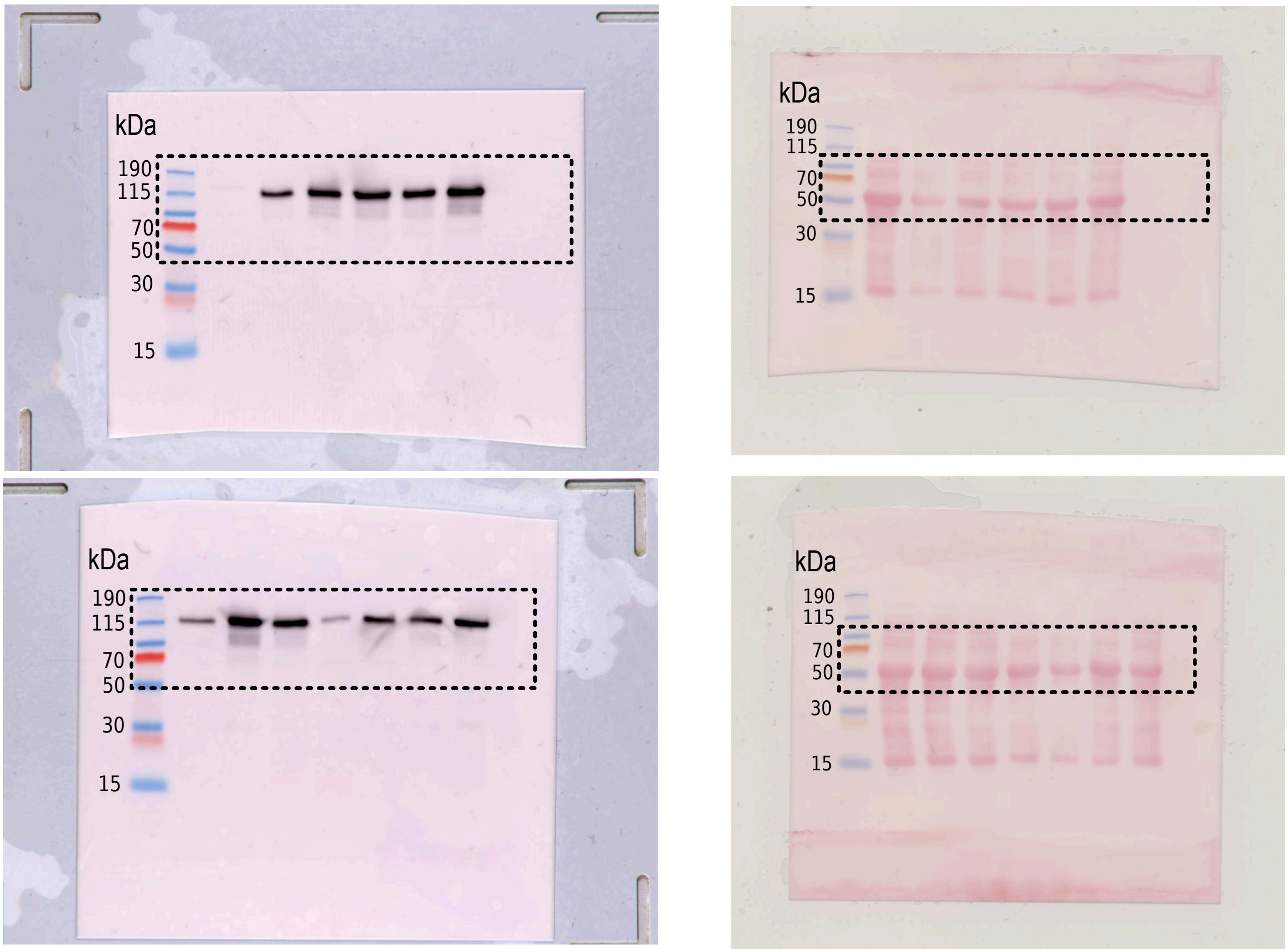

Monoclonal Anti-FLAG-M2-Peroxidase (HRP) antibody (A8592, Merck) (1:5000)
