# Supplementary material for: A small signaling domain controls PPIP5K phosphatase activity in phosphate homeostasis
Source: Nat Commun. 2025 Feb 19;16:1753. doi: 10.1038/s41467-025-56937-0 (PMC11836120; doi:10.1038/s41467-025-56937-0)
Supplement: Supplementary file 2 — Reporting Summary [file 41467_2025_56937_MOESM2_ESM.pdf]

nature portfolio

Corresponding author(s): Michael Hothorn

Last updated by author(s): 2025-13-01

Reporting Summary

Nature Portfolio wishes to improve the reproducibility of the work that we publish. This form provides structure for consistency and transparency in reporting. For further information on Nature Portfolio policies, see our Editorial Policies and the Editorial Policy Checklist.

Statistics

For all statistical analyses, confirm that the following items are present in the figure legend, table legend, main text, or Methods section.

n/a	Confirmed	
☐	☒	The exact sample size (*n*) for each experimental group/condition, given as a discrete number and unit of measurement
☐	☒	A statement on whether measurements were taken from distinct samples or whether the same sample was measured repeatedly
☐	☒	The statistical test(s) used AND whether they are one- or two-sided<br>*Only common tests should be described solely by name; describe more complex techniques in the Methods section.*
☒	☐	A description of all covariates tested
☒	☐	A description of any assumptions or corrections, such as tests of normality and adjustment for multiple comparisons
☐	☒	A full description of the statistical parameters including central tendency (e.g. means) or other basic estimates (e.g. regression coefficient) AND variation (e.g. standard deviation) or associated estimates of uncertainty (e.g. confidence intervals)
☐	☒	For null hypothesis testing, the test statistic (e.g. *F*, *t*, *r*) with confidence intervals, effect sizes, degrees of freedom and *P* value noted<br>*Give P values as exact values whenever suitable.*
☒	☐	For Bayesian analysis, information on the choice of priors and Markov chain Monte Carlo settings
☒	☐	For hierarchical and complex designs, identification of the appropriate level for tests and full reporting of outcomes
☒	☐	Estimates of effect sizes (e.g. Cohen's *d*, Pearson's *r*), indicating how they were calculated

Our web collection on statistics for biologists contains articles on many of the points above.

Software and code

Policy information about availability of computer code

Data collection	X-ray diffraction data sets were collected at the Swiss Light Source (SLS, Villigen, Switzerland) at the beam line X06DA. Negative-stain electron microscopy micrographs were recorded at the DCI-Geneva electron microscopy platform using a Talos L120C microscope (120 kV, Falcon II camera, Thermo Fisher Scientific) using EPU software (version 2.9.0.1519REL). NMR measurements were conducted on a Bruker AV-III spectrometer (Brucker Biospin, Rheinstetten, Germany) with a cryo-QCI probe and operating at 600 MHz for proton nuclei and 151 MHz for carbon nuclei using TOPSPIN (version 3.5). The Malachite Green phosphatase assays were conducted by the quantification of the green complex formation using a Tecan Spark plate reader at 620 nm. Thermal shift data were collected using a QuantStudio 5 Real-Time PCR System (Thermo Fisher Scientific). Rosette phenotype of plants were pictured with a camera Canon EOS250D using the EF-S 18-55 mm lenses. The phosphate quantification in plant extracts was performed using a colorimetric molybdate assay by measuring absorbance at 820 nm with a Tecan Spark plate reader.
Data analysis	Diffraction data were indexed and scaled using XDS (version June 30, 2023). In case of the presence of pseudosymmetry and twinning, the space group was validated using ZANUDA (version 1.0.7). Structures were solved using PHASER (version 2.83). The atomic models were build in COOT (version 0.9.8.93) and refined in PHENIX.REFINE (version 1.21.1-5286). The stereochemistry of the refined models was assessed with PHENIX.MOLPROBITY (version 1.21.1-5286). Structural diagrams were generated with CHIMERA (version 1.17.1-42449). The protein structure comparison was performed on the network service DALI v5 (http://ekhidna2.biocenter.helsinki.fi/dali/). Negative-stain micrographs were analyzed using CryoSPARC (version 4.1). The NMR data were analyzed using TOPSPIN (version 3.5). If the enzymatic reaction curve ended in a linear range, the initial velocity v0 was calculated using the linear regression analysis implemented in GraphPad PRISM (version 10.3.0.507). However, if the NMR spectrometer recorded a hyperbolic reaction curve (meaning that all the substrate has been transformed), the initial velocity v0 was calculated using the one phase decay model implemented in GraphPad PRISM (version 10.3.0.507). The kinetic parameters (v0, Vmax and KM) of the phosphatase activity followed by the Malachite Green assay were calculated using the linear regression analysis and the Michaelis-Menten model implemented in GraphPad PRISM (version 10.3.0.507). Thermal shift assays were analyzed using the

QuantStudio Design & Analysis sofware (version 1.4.3). The rosette area was manually segmented and the area was quantified using the Particle Quantify function implemented in Fiji (imageJ2.14.0/1.54f). The statistical analysis of multiple comparisons between the genotypes and Col-0 were conducted according to Dunnett as implemented in GraphPad PRISM (version 10.3.0.507). The deuterium incorporation levels in HDX-MS experiments were quantified using HD examiner software (version 3.3, Sierra Analytics).

For manuscripts utilizing custom algorithms or software that are central to the research but not yet described in published literature, software must be made available to editors and reviewers. We strongly encourage code deposition in a community repository (e.g. GitHub). See the Nature Portfolio guidelines for submitting code & software for further information.

Data

Policy information about availability of data

All manuscripts must include a data availability statement. This statement should provide the following information, where applicable:

- Accession codes, unique identifiers, or web links for publicly available datasets
- A description of any restrictions on data availability
- For clinical datasets or third party data, please ensure that the statement adheres to our policy

Crystallographic coordinates and associated structure factors have been deposited with the Protein Data Bank (http://rcsb.org) doi: http://doi.org/10.2210/pdb9gr8/pdb (ScVip1KD – ADP, PDB-ID 9GR8), doi: http://doi.org/10.2210/pdb9grh/pdb (ScVip1PD – apo, PDB-ID 9GRH), doi: http://doi.org/10.2210/pdb9grn/pdb (ScVip1PD Δ848-918 – apo, PDB-ID 9GRN) and doi: http://doi.org/10.2210/pdb9gro/pdb (ScVip1PD Δ848-918 RHR-AAA – 1,5-InsP8, PDB-ID 9GRO). The mass spectrometry proteomics data have been deposited to the ProteomeXchange Consortium via the PRIDE partner repository98 with dataset identifier PXD056020 (doi: https://dx.doi.org/10.6019/PXD056020). The source data underlying Figures 3a,b,d,e, 4a-c, 5c-e, 7e,f, 8c,d, 9c,d, 10d,e, and Supplementary Figures S7c, S10b,c are provided as a Source Data file. The Maps and oligonucleotide-based cloning/mutagenesis strategies will be deposit in Addgene under the name of Michael Hothorn. All plasmids used in yeast are available upon request to Prof. Dr. Vikram Govind Panse. All the plant transgenic lines used in this work are stored in our lab and available upon request. The materials will be deposit in the NASC stock center under the name of Michael Hothorn.

Research involving human participants, their data, or biological material

Policy information about studies with human participants or human data. See also policy information about sex, gender (identity/presentation), and sexual orientation and race, ethnicity and racism.

Reporting on sex and gender	No human participants in this study.
Reporting on race, ethnicity, or other socially relevant groupings	No human participants in this study.
Population characteristics	No human participants in this study.
Recruitment	No human participants in this study.
Ethics oversight	No human participants in this study.

Note that full information on the approval of the study protocol must also be provided in the manuscript.

Field-specific reporting

Please select the one below that is the best fit for your research. If you are not sure, read the appropriate sections before making your selection.

[x] Life sciences [ ] Behavioural & social sciences [ ] Ecological, evolutionary & environmental sciences

For a reference copy of the document with all sections, see nature.com/documents/nr-reporting-summary-flat.pdf

Life sciences study design

All studies must disclose on these points even when the disclosure is negative.

Sample size	Since the 1-InsP7, 5-InsP7 and 1,5-InsP8 substrates are not commercially available, the sample size had to be limited to n=1 for the substrate intense NMR measurements, and to n=2 for the malachite green-based enzyme assays, plus a large set of pilot experiments to define the experimental conditions. For plant phenotyping, Pi quantifications and western blotting sample sizes were based on previoulsy published findings (see Zhu et al., 2019 DOI: 10.7554/eLife.43582 ; Ried et al., 2021 DOI: 10.1038/s41467-020-20681-4). For thermal shift protein stability assays n=5 was used according to the manufacturer's instructions (Protein Thermal Shift Dye Kit #4461146, lot no: 2845790, Thermo Scientific).
Data exclusions	No data were excluded.
Replication	All experiments were replicated at least twice, with similar outcome. Structures of ScVip1PD have been determined in three independent crystal lattices, the overall structures are highly similar. NMR and malachite green-based ScVip1PD enzyme assays were reproduced and the two different assays produced similar results. The growth phenotypes, cellular Pi level and AtVIH2 expression levels for our AtVIH2 wild-type and mutant over-expression experiments in Arabidopsis were performed twice (compare Figures 9 and S10).
Randomization	Randomization is not applicable to this structure/function study, which aim at comparing the activity of mutants (in vitro and in vivo) that were selected to specifically target the catalytic site, the substrate binding site or other structurally/functionnally relevant part of the enzyme

identified in the crystal structures.

Blinding: Blinding, which refers to the concealment of group allocation from one or more individuals involved in a clinical research study is not applicable to our enzymatic experiments. Indeed, identifying the target enzyme mutant and the control is important for the technical set-up of these experiments.

Reporting for specific materials, systems and methods

We require information from authors about some types of materials, experimental systems and methods used in many studies. Here, indicate whether each material, system or method listed is relevant to your study. If you are not sure if a list item applies to your research, read the appropriate section before selecting a response.

Materials & experimental systems

n/a	Involved in the study
☐	☒ Antibodies
☐	☒ Eukaryotic cell lines
☐	☐ Palaeontology and archaeology
☐	☐ Animals and other organisms
☐	☐ Clinical data
☐	☐ Dual use research of concern
☐	☒ Plants

Methods

n/a	Involved in the study
☐	☐ ChIP-seq
☐	☐ Flow cytometry
☐	☐ MRI-based neuroimaging

Antibodies

Antibodies used: Anti-His6-Peroxidase, REF 11 965 085 001 (Merck/Sigma-Aldrich), Mouse Monoclonal antibody (clone BMG-His-1), LOT 16830100. (dilution 1:3000)

Monoclonal ANTI-FLAG® M2-Peroxidase (HRP) antibody produced in mouse, REF A8592-5X1MG (Merck/Sigma-Aldrich), clone M2, LOT # SLBH1183V. (dilution 1:5000)

Validation: According to the manufacturer's website, Anti-His6-Peroxidase targets His6-tagged proteins (https://www.sigmaaldrich.com/CH/en/product/roche/11965085001), and Monoclonal ANTI-FLAG binds to FLAG fusion proteins and recognizes the FLAG epitope at N-terminal, Met-N-terminal, C-terminal, and internal FLAG peptides https://www.sigmaaldrich.com/CH/en/product/sigma/a8592).

Eukaryotic cell lines

Policy information about cell lines and Sex and Gender in Research

Cell line source(s): Trichoplusia ni BTI-Tnao38 cells (Boyce Thompson Institute, #160776, Ximbio) using recombinant baculoviruses (Hashimoto, Y., Zhang, S., & Blissard, G. W., 2010)

The yeast wild type strains used in this study are BY4741 (Y00000) background (Euroscarf).

Authentication: Not applicable.

Mycoplasma contamination: Not tested for mycoplasma contamination.

Commonly misidentified lines (See ICLAC register): Not applicable.

Palaeontology and Archaeology

Specimen provenance: *Provide provenance information for specimens and describe permits that were obtained for the work (including the name of the issuing authority, the date of issue, and any identifying information). Permits should encompass collection and, where applicable, export.*

Specimen deposition: *Indicate where the specimens have been deposited to permit free access by other researchers.*

Dating methods: *If new dates are provided, describe how they were obtained (e.g. collection, storage, sample pretreatment and measurement), where they were obtained (i.e. lab name), the calibration program and the protocol for quality assurance OR state that no new dates are provided.*

☐ Tick this box to confirm that the raw and calibrated dates are available in the paper or in Supplementary Information.

Ethics oversight	*Identify the organization(s) that approved or provided guidance on the study protocol, OR state that no ethical approval or guidance was required and explain why not.*

Note that full information on the approval of the study protocol must also be provided in the manuscript.

Animals and other research organisms

Policy information about studies involving animals; ARRIVE guidelines recommended for reporting animal research, and Sex and Gender in Research

Laboratory animals	*For laboratory animals, report species, strain and age OR state that the study did not involve laboratory animals.*
Wild animals	*Provide details on animals observed in or captured in the field; report species and age where possible. Describe how animals were caught and transported and what happened to captive animals after the study (if killed, explain why and describe method; if released, say where and when) OR state that the study did not involve wild animals.*
Reporting on sex	*Indicate if findings apply to only one sex; describe whether sex was considered in study design, methods used for assigning sex. Provide data disaggregated for sex where this information has been collected in the source data as appropriate; provide overall numbers in this Reporting Summary. Please state if this information has not been collected. Report sex-based analyses where performed, justify reasons for lack of sex-based analysis.*
Field-collected samples	*For laboratory work with field-collected samples, describe all relevant parameters such as housing, maintenance, temperature, photoperiod and end-of-experiment protocol OR state that the study did not involve samples collected from the field.*
Ethics oversight	*Identify the organization(s) that approved or provided guidance on the study protocol, OR state that no ethical approval or guidance was required and explain why not.*

Note that full information on the approval of the study protocol must also be provided in the manuscript.

Clinical data

Policy information about clinical studies

All manuscripts should comply with the ICMJEguidelines for publication of clinical research and a completedCONSORT checklist must be included with all submissions.

Clinical trial registration	*Provide the trial registration number from ClinicalTrials.gov or an equivalent agency.*
Study protocol	*Note where the full trial protocol can be accessed OR if not available, explain why.*
Data collection	*Describe the settings and locales of data collection, noting the time periods of recruitment and data collection.*
Outcomes	*Describe how you pre-defined primary and secondary outcome measures and how you assessed these measures.*

Dual use research of concern

Policy information about dual use research of concern

Hazards

Could the accidental, deliberate or reckless misuse of agents or technologies generated in the work, or the application of information presented in the manuscript, pose a threat to:

No	Yes	
☒	☐	Public health
☒	☐	National security
☒	☐	Crops and/or livestock
☒	☐	Ecosystems
☒	☐	Any other significant area

Experiments of concern

Does the work involve any of these experiments of concern:

No	Yes	
☒	☐	Demonstrate how to render a vaccine ineffective
☒	☐	Confer resistance to therapeutically useful antibiotics or antiviral agents
☒	☐	Enhance the virulence of a pathogen or render a nonpathogen virulent
☒	☐	Increase transmissibility of a pathogen
☒	☐	Alter the host range of a pathogen
☒	☐	Enable evasion of diagnostic/detection modalities
☒	☐	Enable the weaponization of a biological agent or toxin
☒	☐	Any other potentially harmful combination of experiments and agents

Plants

Seed stocks

All the transgenic lines used in this assay are stored in our lab. The materials will be deposit in the NASC stock center under the name of Michael Hothorn.

Novel plant genotypes

12 novel transgenic lines were generated, proUBI10::AtVIH2WT::Flag, proUBI10::AtVIH2K219A-D292A::Flag, proUBI10::AtVIH2R372A-H373A::Flag, proUBI10::AtVIH2R372A-H373A-R376A::Flag, proUBI10::AtVIH2R372A-H373A-R376A-E882A::Flag, proUBI10::AtVIH2H741A::Flag, proUBI10::AtVIH2C696A::Flag, proUBI10::AtVIH2H505A-H741A-H975A::Flag, proUBI10::AtVIH2P378V-G500V-G501A::Flag, proUBI10::AtVIH2R722S-D724A-T726A::Flag, proUBI10::AtVIH2K383A-K447A-K598A-R722A::Flag and proUBI10::AtVIH2K379A-K381A-K498A::Flag.

Authentication

The vectors containing the AtUBI10 promoter (proUBI10), the corresponding AtVIH2 coding sequence, a FLAG tag, the Nos terminator and Fast Red as a fluorescent marker for the seed coat were transformed into A. tumefaciens strain GV3101. Flower dipping was then used to transform 5-week-old plants (Col-0). T1 plants were selected by red fluorescence using a Nikon SMZ18 stereo-microscope equipped with an RFP-B filter. Lines with single T-DNA insertions were selected by the segregation analysis. T3 lines were propagated and harvested for the seed donation. The published lines will display a 100% of seed showing the RFP in the seed coat and the protein expression can be detected with the anti-flag antibody showing a band around 118 Kda.

ChIP-seq

Data deposition

☐ Confirm that both raw and final processed data have been deposited in a public database such as GEO.

☐ Confirm that you have deposited or provided access to graph files (e.g. BED files) for the called peaks.

Data access links
May remain private before publication.

For "Initial submission" or "Revised version" documents, provide reviewer access links. For your "Final submission" document, provide a link to the deposited data.

Files in database submission

Provide a list of all files available in the database submission.

Genome browser session
(e.g. UCSC)

Provide a link to an anonymized genome browser session for "Initial submission" and "Revised version" documents only, to enable peer review. Write "no longer applicable" for "Final submission" documents.

Methodology

Replicates

Describe the experimental replicates, specifying number, type and replicate agreement.

Sequencing depth

Describe the sequencing depth for each experiment, providing the total number of reads, uniquely mapped reads, length of reads and whether they were paired- or single-end.

Antibodies

Describe the antibodies used for the ChIP-seq experiments; as applicable, provide supplier name, catalog number, clone name, and lot number.

Peak calling parameters

Specify the command line program and parameters used for read mapping and peak calling, including the ChIP, control and index files used.

Data quality

Describe the methods used to ensure data quality in full detail, including how many peaks are at FDR 5% and above 5-fold enrichment.

Software

Describe the software used to collect and analyze the ChIP-seq data. For custom code that has been deposited into a community repository, provide accession details.

Flow Cytometry

Plots

Confirm that:

- [ ] The axis labels state the marker and fluorochrome used (e.g. CD4-FITC).
- [ ] The axis scales are clearly visible. Include numbers along axes only for bottom left plot of group (a 'group' is an analysis of identical markers).
- [ ] All plots are contour plots with outliers or pseudocolor plots.
- [ ] A numerical value for number of cells or percentage (with statistics) is provided.

Methodology

Sample preparation	*Describe the sample preparation, detailing the biological source of the cells and any tissue processing steps used.*
Instrument	*Identify the instrument used for data collection, specifying make and model number.*
Software	*Describe the software used to collect and analyze the flow cytometry data. For custom code that has been deposited into a community repository, provide accession details.*
Cell population abundance	*Describe the abundance of the relevant cell populations within post-sort fractions, providing details on the purity of the samples and how it was determined.*
Gating strategy	*Describe the gating strategy used for all relevant experiments, specifying the preliminary FSC/SSC gates of the starting cell population, indicating where boundaries between "positive" and "negative" staining cell populations are defined.*

- [ ] Tick this box to confirm that a figure exemplifying the gating strategy is provided in the Supplementary Information.

Magnetic resonance imaging

Experimental design

Design type	*Indicate task or resting state; event-related or block design.*
Design specifications	*Specify the number of blocks, trials or experimental units per session and/or subject, and specify the length of each trial or block (if trials are blocked) and interval between trials.*
Behavioral performance measures	*State number and/or type of variables recorded (e.g. correct button press, response time) and what statistics were used to establish that the subjects were performing the task as expected (e.g. mean, range, and/or standard deviation across subjects).*

Acquisition

Imaging type(s)	*Specify: functional, structural, diffusion, perfusion.*
Field strength	*Specify in Tesla*
Sequence & imaging parameters	*Specify the pulse sequence type (gradient echo, spin echo, etc.), imaging type (EPI, spiral, etc.), field of view, matrix size, slice thickness, orientation and TE/TR/flip angle.*
Area of acquisition	*State whether a whole brain scan was used OR define the area of acquisition, describing how the region was determined.*

Diffusion MRI ☐ Used ☐ Not used

Preprocessing

Preprocessing software	*Provide detail on software version and revision number and on specific parameters (model/functions, brain extraction, segmentation, smoothing kernel size, etc.).*
Normalization	*If data were normalized/standardized, describe the approach(es): specify linear or non-linear and define image types used for transformation OR indicate that data were not normalized and explain rationale for lack of normalization.*
Normalization template	*Describe the template used for normalization/transformation, specifying subject space or group standardized space (e.g. original Talairach, MNI305, ICBM152) OR indicate that the data were not normalized.*
Noise and artifact removal	*Describe your procedure(s) for artifact and structured noise removal, specifying motion parameters, tissue signals and physiological signals (heart rate, respiration).*

Volume censoring | *Define your software and/or method and criteria for volume censoring, and state the extent of such censoring.*

Statistical modeling & inference

Model type and settings | *Specify type (mass univariate, multivariate, RSA, predictive, etc.) and describe essential details of the model at the first and second levels (e.g. fixed, random or mixed effects; drift or auto-correlation).*

Effect(s) tested | *Define precise effect in terms of the task or stimulus conditions instead of psychological concepts and indicate whether ANOVA or factorial designs were used.*

Specify type of analysis: ☐ Whole brain ☐ ROI-based ☐ Both

Statistic type for inference | *Specify voxel-wise or cluster-wise and report all relevant parameters for cluster-wise methods.*

(See Eklund et al. 2016)

Correction | *Describe the type of correction and how it is obtained for multiple comparisons (e.g. FWE, FDR, permutation or Monte Carlo).*

Models & analysis

n/a	Involved in the study
☐	☐ Functional and/or effective connectivity
☐	☐ Graph analysis
☐	☐ Multivariate modeling or predictive analysis

Functional and/or effective connectivity | *Report the measures of dependence used and the model details (e.g. Pearson correlation, partial correlation, mutual information).*

Graph analysis | *Report the dependent variable and connectivity measure, specifying weighted graph or binarized graph, subject- or group-level, and the global and/or node summaries used (e.g. clustering coefficient, efficiency, etc.).*

Multivariate modeling and predictive analysis | *Specify independent variables, features extraction and dimension reduction, model, training and evaluation metrics.*
